# Clinical efficacy of titanium prepared platelet rich fibrin in periodontal regeneration: A systematic review and meta-analysis

**DOI:** 10.12688/f1000research.131461.2

**Published:** 2024-05-24

**Authors:** Dr. Ranu Oza, Dr. Prasad Dhadse, Dr. Pavan Bajaj, Dr. Komal Bhombe, Dr. Khushboo Durge, Dr. Chitrika Subhadarsanee, Dr. Safiya Hassan

**Affiliations:** 1 Post Graduate student, Department of Periodontics and Implantology, Sharad Pawar Dental College and Hospital, Datta Meghe Institute of Medical Sciences, Wardha, Maharashtra, 442001, India; 2 Professor and Head of the Department, Department of Periodontics and Implantology, Sharad Pawar Dental College and Hospital, Datta Meghe Institute of Medical Sciences, Wardha, Maharashtra, 442001, India; 3 Reader, Department of Periodontics and Implantology, Sharad Pawar Dental College and Hospital, Datta Meghe Institute of Medical Sciences, Wardha, Maharashtra, 442001, India; 4 MDS, Department of Periodontics and Implantology, Sharad Pawar Dental College and Hospital, Datta Meghe Institute of Medical Sciences, Wardha, Maharashtra, 442001, India; 5 Assistant Professor, Department of Periodontics and Implantology, Sharad Pawar Dental College and Hospital, Datta Meghe Institute of Medical Science, Wardha, Maharashtra, 442001, India

**Keywords:** T-PRF, Platelet Concentrates, Chronic Periodontitis, Infrabony Defects, Systematic Review, Meta-analysis

## Abstract

**Background:**

Periodontal regeneration therapies frequently involve autologous platelet concentrates (APCs). They can be used in sinus lift surgeries and socket preservation, among other clinical settings. Platelet rich fibrin (PRF) membrane has been used to treat gingival recession in individuals or groups of individuals using a coronally progressed or lateral pedicle flap. In the treatment of mixed periodontic endodontic lesion/furcation defect, PRF functions as a healing and interpositional biomaterial, filling a cystic cavity. PRF is known to help the bone regeneration process. In the last few years, efforts have been made to enhance the PRFs characteristics and quality. One of them is titanium platelet rich fibrin (T-PRF). Third-generation platelet concentrate no longer contains silica, and its preparation in glass vacuum containers, that no longer creates any known concerns. The effectiveness PRF’s has been evaluated in connective tissue and bone repair. The aim of this study is to compare T-PRF to other platelet concentrates and different treatment modalities for periodontal regenerative procedures.

**Methods:**

A protocol of this systematic review have been registered in prospero (CRD42022293545). The online database searched were PUBMED, COCHRANE for published articles up to November 2022 without language restrictions. Studies in trial registers, handsearching, bibliographic references of relevant articles were also checked. Data collection and analysis was done by individual authors. Independent eligibility assessments were conducted by four review authors. Then, using the standard Cochrane methodology, four review authors extracted the data and evaluated the risk of bias for individual studies. We developed “Summary of findings” tables and used GRADE to evaluate the evidence.

**Results:**

Three studies were included for meta-analysis. Results of meta-analysis supported that T-PRF is effective for correction of both hard and soft tissue defects.

**Conclusions:**

The overall qualitative and quantitative analysis suggest that T-PRF has superior structural properties and thicker fibrin network for ensuring predictable success periodontal regenerative procedures.

## Introduction

### Description of the condition

Plasma fractions with higher platelet concentrations, such as platelet-rich plasma (PRP) and platelet rich fibrin (PRF) are crucial to regeneration. ^1^ Their use has been successfully linked to connective tissue and bone repair. ^2^ PRP has been documented and used in a variety of applications with what appears to be clinical success. It is an autologous modification of fibrin glue that is created by techniques that concentrate autologous platelets. ^1^ PRP supports bioactivity but exhibits no stimulant properties for activation of regenerative cells. ^3^ Additionally, its preparation is technique sensitive. ^4^ PRP preparation, on the other hand, takes time therefore various efforts have been made to modify and enhance the PRP’s characteristics and quality which include Leukocyte platelet-rich fibrin (L-PRF). It was first described by Choukroun as cited by Dohan DM *et al*. (2006). ^5^ It is regarded as a second-generation platelet concentrate and has been used to speed up wound healing during several surgical operations. Choukroun *et al.* first created PRF in France in 2001 as an autologous biomaterial that includes leucocytes. ^6^ The method does not require bovine thrombin or an anticoagulant, in contrast to other platelet-rich products (or any other gelling agent). ^5^^,^^7^^–^^9^ Although PRF has been shown to produce positive therapeutic outcomes, some clinicians are concerned that the method’s use of glass-evacuated collection tubes for blood with silica activators may pose a health risk. ^10^^,^^11^ O’Connell provided a description of the inevitable silica interaction. ^12^ Although the silica in the tube is thick enough to settle with the red blood cells, some of the particles are still colloidally suspended in the buffy coat, fibrin, and platelet-poor layers of plasma. Therefore, when the product is used for therapy, the particles could come in contact with the patient. ^13^ Silica, found in glass test tubes, is known for its ill effects. However, T-PRF, a third-generation platelet concentrate, is recognized for its ability to mitigate this problem.

### Description of the intervention

Nonsurgical periodontal therapy, such as scaling and root planing (SRP) alone or SRP plus systemic or local anti-inflammatory or antibacterial drugs, to surgical flap debridement, are the approaches for treatment of periodontitis cases. ^14^ However, in cases of soft and hard tissue defects, surgical correction of the defects is of prime importance. For correction of bony defects, the use of autografts and allografts have been advocated. ^15^ Autologous platelet concentrates when used in conjunction with bone grafts results in better improvement. A study by Olgun *et al*. (2018) reported clinical, radiographic and histological outcomes of bone that accelerated to four months compared to six months for allografts when combined with T-PRF. ^16^ The desired outcome was seen in four months as compared to six months. Tunalı *et al.* in the year 2012 ^17^ discovered a mature fibrin network when T-PRF clots were examined. In the T-PRF membrane, they discovered islets of bone tissue and newly developing connective tissue. These findings demonstrate that T-PRF could, within 30 days of treatment, cause the development of new bone and connective tissue in an experimental rabbit model of wound healing.

### How the intervention might work

As mentioned above, the novel product termed T-PRF was prepared by Tunali *et al*. (2014) ^13^ utilising a modified L-PRF process. Authors took the advantage of Dohan Ehrenfest’s taxonomy, which divides platelet-rich products into four main groups to prevent any misunderstanding about how to obtain these products. These four categories are determined according to the number of leukocytes and fibrin they contain, platelet-rich products: just platelet-rich plasma products, platelet- and leukocyte rich plasma products, platelet-rich fibrin, and platelet and leukocyte rich fibrin. The creation of T-PRF, a new platelet concentration, was motivated by the idea that titanium tubes would be more potent at activating platelets than the glass tubes used in Chouckron’s approach. The distinguishing properties of T-PRF, particularly its improved biocompatibility, are produced by platelet activation with titanium as opposed to activation with silica particles. ^18^ T-PRF fibrin network covers a larger area than L-PRF fibrin network, also fibrin seemed thicker in the T-PRF samples. ^13^

### Preparation of Titanium Platelet Rich Fibrin (T-PRF)

Immediately before the procedure, intravenous blood is withdrawn into a 10 ml sterile titanium test tube without the use of an anticoagulant. The tubes are immediately spun in a centrifuge for 12 minutes at 2700 rpm. The creation of a structured fibrin clot in the middle of the tube, in between the red corpuscles at the bottom and the acellular plasma [Platelet Poor Plasma (PPP)] at the top, is made possible by centrifuging blood as soon as it is collected. The titanium is very active surface for coagulation. Hence, centrifugation is started in 30-60 seconds. After centrifugation the T-PRF is kept inside the tube for 10-15 minutes to keep activation process in continuation.

### Why it is important to do this review

Since the introduction of T-PRF by Tunali *et al.*, ^18^ it has been assessed for various properties including its structural and biochemical characteristics. Tunalı *et al.* (2014) ^19^ described the structural properties of T-PRF, and compared it to L-PRF. T-PRF samples appeared to have a highly ordered network with continuous integrity. The fibrin network in T-PRF samples appeared to be thicker and to cover a wider area than the network in L-PRF samples, according to histomorphometric analyses. T-PRF is a naturally occurring leukocyte- and PRF product that has been defined for the first time in a human investigation. Titanium platelet activation appears to provide T-PRF with certain high features. Since then T-PRF has been used for various periodontal procedures including treatment of various infrabony defects, gingival recession defects, in open flap debridement cases, periimplantitis. ^13^^,^^16^^,^^18^^,^^20^^–^^22^

Systematic evaluation of the studies to assess the clinical efficacy have not been done to date. Therefore the objective of this systematic review is provide the best possible evidence for the use of T-PRF in periodontal regenerative procedures.

## Methods

Protocol Development Update: This review protocol has been officially registered in Prospero under the registration number CRD42022293545. To assess the methodological quality of this systematic review, we have adopted the PRISMA (Preferred Reporting Items for Systematic Reviews and Meta-Analyses) statement. This tool, available at www.prisma-statement.org/, serves as a guide for evaluating systematic reviews. It builds upon the original QUOROM guideline and facilitates the assessment of systematic reviews encompassing both randomized and non-randomized controlled trials.

Focused Question: The primary inquiry of this systematic review is as follows: What is the efficacy of Titanium Platelet Rich Fibrin in periodontal regenerative procedures for patients diagnosed with chronic periodontitis, as observed in adults aged 18 years and older, based on the collective evidence derived from existing literature encompassing both randomized and non-randomized clinical trials? We searched PubMed, Cochrane, Embase database without language restrictions. We used Medical subject headings (MeSH) or equivalent and textword terms. We searched the metaRegister of controlled trials ( mRCT), National clinical trials government website. Additionally, we checked the reference lists of reviews, retrieved articles for additional studies, and performed citation searches on key articles manually by going through each reference of the included articles. We intended to use randomised controlled trials (RCTs) that assessed outcomes in an open or blinded manner. Short abstracts (typically meeting reports), non-randomised research, experimental pain studies, animal model studies, case reports, and clinical observational studies were all omitted.

*Search strategy*

(“t prf”[Title/Abstract] OR “titanium prepared platelet rich fibrin”[Title/Abstract] OR ((“titanium”[MeSH Terms] OR “titanium”[All Fields] OR “titaniums”[All Fields]) AND “platelet rich fibrin”[MeSH Terms])) AND (“gingival recession”[Title/Abstract] OR “gingival recession”[MeSH Terms] OR “furcation defects”[MeSH Terms] OR “alveolar ridge augmentation”[MeSH Terms] OR “infrabony defect”[Title/Abstract] OR “furcation”[Title/Abstract] OR “periimplantitis”[Title/Abstract])

Systemically healthy patients (18+ years) clinically diagnosed with any type of periodontal disease (infrabony defects, gingival recession, osseous defects) were included irrespective of gender and race). Our outcome measures included change in the indices [Plaque index (PI) Gingival index (GI)], mean of periodontal pocket depth (PPD), mean of relative vertical clinical attachment loss (RVCAL), mean of relative horizontal clinical attachment loss (RHCAL) and mean of bone defect (BD). All the parameters were measured at baseline, 3 months and 6 months.

### Data collection and analysis

*Selection of studies*

**Figure 1.**
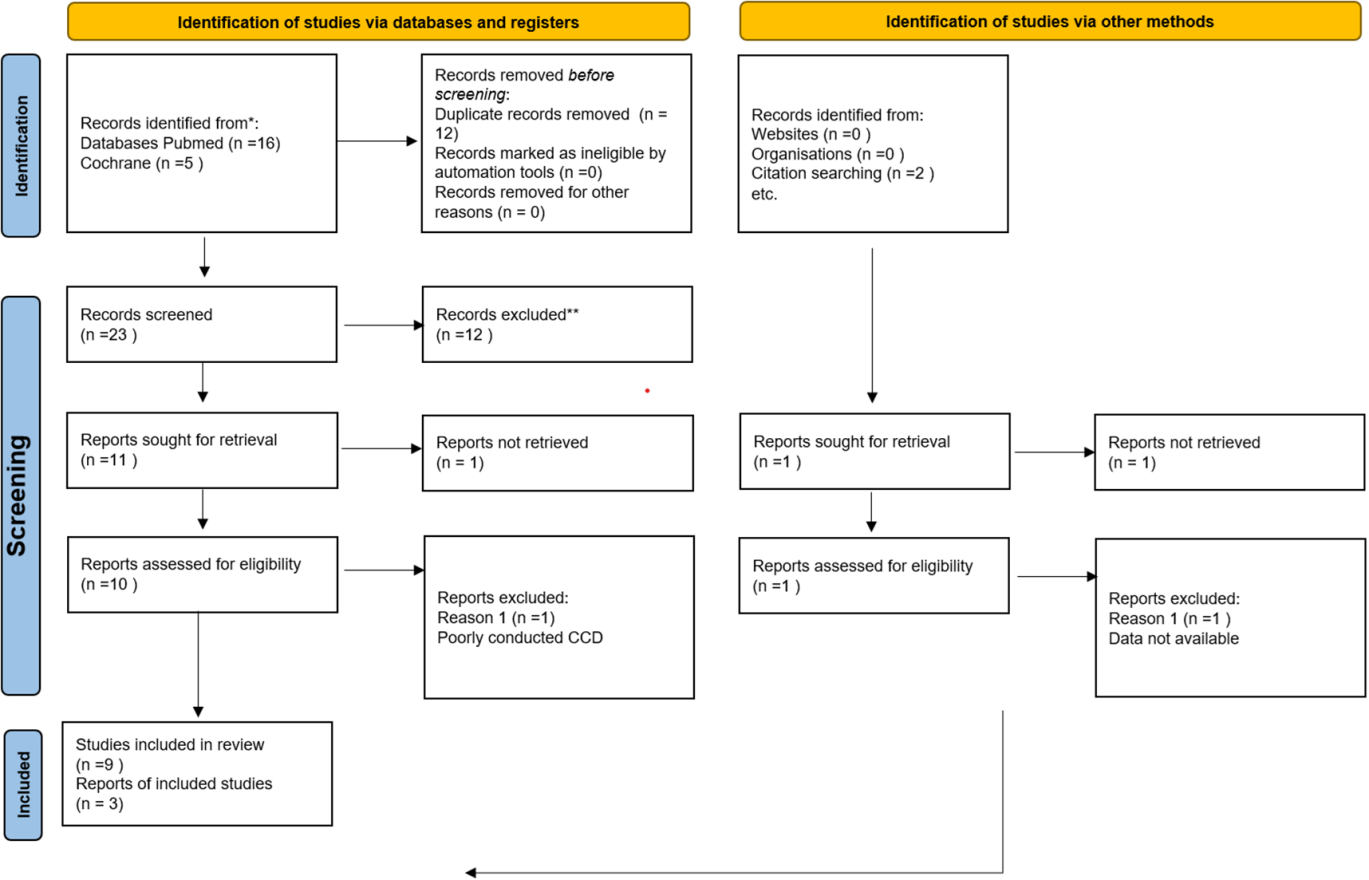
PRISMA 2020 flow diagram.

An open source online screening tool Rayyan ^23^ (RRID:SCR_017584) was used by review writers. They independently screened the search results (RO, PD, PB, KB). By reading through the abstracts of each study that the search produced, the eligibility of each research was established. The review writers excluded studies that did not distinctly meet the inclusion criteria. For all included studies’ complete texts were acquired. Primary reviewers independently screened the complete texts of these studies to pick the most pertinent studies (RO, PD, PB, KB). The respective authors were reached by phone or email to get the required clarification for any missing data or information in the studies that had an impact on the study selection criteria. In cases of disagreement or dispute, a fifth author was asked for a judgement (KD). Prior to evaluation, the trials were not anonymized. Any language restrictions in the study selection process were not taken into consideration as a constraint for carrying out this evaluation. The complete review includes a PRISMA flow chart ^32^ that shows the precise status of all identified studies in accordance with the recommendation found in “Part 2, Section 11.2.1 of the Cochrane Guidelines for Systematic Reviews of Interventions” *.* ^24^ Irrespective of the reporting of outcome data, studies were included in this review as shown in Figure 1.

### Data extraction and management

The data extraction from “included studies” was performed by four reviewers (RO, CS, KB, and KD) and given in the “characteristics of studies table” using a pre-defined data extraction form ( Table 1). Data were extracted based on the nature of research, participant information, intervention information, and reported results. The disagreement between the main reviewers was resolved by the third reviewer (PB). The fourth reviewer successfully addressed the “risk of bias evaluation” discrepancy (KB). We have included a total of nine studies for qualitative analysis and have been included in the characteristics of studies table ( Table 1). All the studies were matched for type of intervention, type of defect and participant details. Three studies were included and matched for meta-analysis followed by quantitative analysis. ^20^^,^^22^^,^^25^

**Table 1.** Characteristics of studies.

Articles	Taner Arabaci *et al.*, 2018	Shiva Shankar Gummaluri *et al.*, 2020	Gülbahar Ustaoğlu *et al.*, 2020	HG Pirebas *et al.*, 2022	Ebru Olgun *et al.*, 2018	Gülbahar Ustaoğlu, *et al*, 2016	Gülbahar Ustaoğlu, *et al.*, 2019	Gülbahar Ustaoğlu, *et al.*, 2020	Bilge Cansu Uzun *et al.*, 2017
**Country**	Turkey	Uttar Pradesh, India	Turkey	Turkey	Turkey	Turkey	Turkey	Turkey	Turkey
**Type of study**	RCT	RCT	RCT	RCT	RCT	RCT	RCT	RCT	RCT
**Details of group**									
Number of groups	Two (group 1: T-PRF+OFD), (Group 2: OFD alone)	Two (group 1: T-PRF, group 2: L-PRF)	3 (Group 1: T-PRF, Group 2: GTR, Group 3: OFD)	1 (Test side: T-PRF+allograft, Control side: allograft)	2 (Group 1: T-PRF, Group 2: Allograft)	2 (Group 1: T-PRF, Group 2: Untreated)	3 (Group 1: L-PRF, Group 2: T-PRF, Group 3: Natural healing)	2 (Group 1: T-PRF, Group 2: CTG)	2 (Group 1: T-PRF, Group 2: CTG)
Number of patients	29 subjects	26 subjects	45 patients	25 subjects	18 subjects	40 subjects	57 subjects	30 subjects	34 subjects (115 recession defects)
Number of males and females in the group	17 males/12 females	NR	23males/22females	9males/16females	9males/9females	NR	28males/29females	NR	NR
Number of patients completed study	NR	26 subjects	NR	25 subjects	18 subjects	NR	NR	NR	NR
**Participation details**									
Age	28-49 years	20-55 years	26–59 years	26–59 years	42–69 years	NR	18+ years	18+ years	18+ years
Type of periodontal disease	Chronic Periodontitis	3- walled infrabony defects	IBDs with endo perio involvement	chronic periodontitis	chronic periodontitis	Free gingival graft	Extraction socket healing	Peri-implant soft tisssue thickness	Gingival Recession
Tooth mobility	NR	NR	NA	NR	NR	NR	NR	NR	NR
**Intervention**									
Type	SRP+OFD+T-PRF	OFD+T-PRF	OFD+T-PRF & OFD+GTR	T-PRF+allograft	T-PRF+allograft	T-PRF	T-PRF	T-PRF	T-PRF
Number of sites	one quadrant per patient	one intrabony defect of ≥3 mm	IBDs associated with primary periodontal lesion with secondary endodontic involvement or true combined endodontic- periodontal lesions in single-rooted teeth	One site per patient	residual crest height of <5 mm in the posterior maxilla	NA	NA	Thin biotype<2mm	Miller class I or II teeth among mandibular or maxillary incisors and premolars.
Duration	9 months	9 months	9 months	45 days	9 months	9 months	2 weeks	3 months	12 months
Frequency	baseline, 2weeks, 4 weeks, 6 weeks, 9months.	baseline, 3 month, 6months, 9 months	baseline, 9 months	3rd, 7th, 14th, and 30th days	baseline, 7/9months	baseline, 3,7,21 days/PSTT: Baseline, 9 months	baseline, 3, 1st day, 1 week, 2 weeks	baseline, 3 months	baseline, 6 months, 12 months
**Outcomes**									
Name	primary- GCF concentrations of FGF-2, PDGF-BB, and TGF-β1,relative receptor activator nuclear factor kappa-B/osteoprotegerin (RANKL/OPG)	primary-PD,PI,GI, CAL	Primary: PD, CAL, and radiographic IBD	Primary: PDGF-BB, VEGF-A, FGF-2, ANG, ANT	Primary: Implant stability, Bone density, Newly formed bone, Cancellous bone ratio, Bone volume, Bone height.	Primary: colour match, H2O2-bubbling tests for CWE,PSTT	Primary: H2O2-bubbling tests for CWE, FD, VAS,LWHI	Primary: KTW, peri-implant STT	Primary: KTW, Clinical periodontal indexes, gingival thickness, and recession depth
	secondary- PD, RAL, GML	NR	NA	NA	NA	NA	NA	NA	NA
Criteria for measurement	PD≥ 5mm,moderate: 3-4 mm CAL; severe: ≥5 mm CAL	CAL ≥3 mm PPD ≥5 mm in more than two teeth	PD ≥ 5 mm with two- or three-wall IBDs ≥ 3 mm deep	two- or three IBDs ≥3 mm deep+ interproximal PD ≥5 mm	NA	NA	NA	NA	NA
Reference point and method used	A custom made acrylic stent for RVCAL,occlusal surface used as reference point.	A custom made acrylic stent for RALs,occlusal surface used as reference point.	A custom made acrylic stent for RALs,occlusal surface used as reference point.	NR	NA	NA	NA	NA	NA
Time of reporting	9 months	3 months	9 months	30 days	9 months	9 months	2 weeks	9 months	12 months

**Abbreviations:** RCT - Randomized controlled trials; SRP - Scaling and root planning; OFD - Open flap debridement; GTR - Guided tissue regeneration; PPD - Probing pocket depth; DF - Defect fill; RVCAL - Relative vertical clinical attachment loss; RHCAL - Relative horizontal clinical attachment loss; NR - Not reported; CHX - Chlorhexidine; NA - Not applicable; PI - Plaque index; GI - Gingival index; CEJ - Cemento-enamel junction.

### Assessment of risk of bias in included studies

Four reviewers (RO, PD, CS, and SH) from each included study independently evaluated the risk of bias (RoB) using the Cochrane domain-based, two-part tool as outlined in Chapter 8 of the Cochrane Handbook for Systematic Reviews of Interventions. ^24^ The fourth reviewer was able to resolve the disagreement between the main reviewers (KB). Sequence generation, allocation concealment, participant and staff blinding, blinding of outcome evaluation, incomplete outcome data, biased outcome reporting, and other bias, such as baseline imbalance, were all areas in which we evaluated the RoB.

### Measures of treatment effect

In parallel-group RCTs, the unit of analysis was the individual subject. The Elbourne-recommended method is used to integrate the cross-over designed trials into the meta-analysis. ^26^ Measurements from experimental intervention periods and control intervention periods, respectively, were taken for these trials, and they were analysed under the assumption that it was a parallel group study of intervention versus control.

### Assessment of heterogeneity

The Chi ^2^ test (P value set at 0.10 for statistical significance) was used to examine clinical heterogeneity, and the I ^2^ statistic was utilised to quantify heterogeneity in the outcomes of the included studies using RevMan manager version 5.0. ^27^ Significant heterogeneity is defined as I ^2^ over 75%; substantial heterogeneity is defined as I ^2^ between 50% and 90%; moderate heterogeneity is defined as I ^2^ between 30% and 60%; and mild heterogeneity is defined as I ^2^ less than 40%. If statistical heterogeneity with I ^2^ more than or equal to 50% is found, relevant causes were investigated using predefined subgroup analysis, and a random-effects model was used and reported.

### Data synthesis

Only when it was determined that the participants, interventions, comparisons, and results of the included studies were adequately comparable to yield a conclusion with clinical relevance and significance was a meta-analysis performed. We intended to carry out the meta-analysis using the Cochrane Collaboration’s open source RevMan 2014 statistical software (RRID: SCR_003581). If statistical heterogeneity with I ^2^ higher than or equal to 50% is found, the sources of the heterogeneity were found, and a random-effects model meta-analysis was then carried out. In a meta-analysis, four papers were used. Table 2 presents data from all three investigations (Data Entry).

**Table 2.** Data entry.

Parameters	Arabaci 2018		Shiva Shankar Gummaluri 2020		Gülbahar Ustaoğlu 2020	
**Outcomes**	Continuous data		Continuous data		Continuous data	
	Mean of intervention±standard deviation (ofd+t-prf)	Mean of control ±standard deviation (ofd)	Mean of intervention±standard deviation (flap+t-prf)	Mean of control±standard deviation (flap+l-prf)	Mean of intervention±standard deviation (flap+t-prf)	Mean of control±standard deviation (flap)
**Plaque index (PI)**	Baseline (Bl) (n=29): 0.51±0.14 9 months (n=29): 0.55±0.22	Bl (n=29): 0.49±0.14 9 months (n=29): 0.58±0.21	Bl (n=18): 1.74±0.34 9 month (n=17): 0.45±0.36	Bl (n=18):1.79±0.5 9 months (n=17): 0.71±0.38	Not applicable (NA) N=15	NA N=15
**Gingival index (GI)**	Not reported (NR)	NR	Bl: 1.71±0.44 9 months: 0.26±0.14	Bl:1.46±0.48 9 months: 0.30±0.16	NA	NA
**Probing pocket depth (PPD)**	Bl: 8.15±1.75 9 month: 4.01±1.31	Bl: 8.11±1.98 9 month: 5.14±1.29	BL:8.65±1.50 9 months: 4.82±1.78	Bl:7 8.18±1.29 9 months: 3.29±1.1	Bl: 9.15±1.65 9 months: 4.46±0.90	Bl: 9.85±1.69 9 months: 4.17±1.56
**Clinical attachment loss (CAL)**	Bl: 7.49±1.41 9 month: 3.82±0.87	Bl: 7.37±1.18 9 month: 5.05±0.99	Bl: 9.06±1.56 9 months: 4.12±1.58	Bl: 8.59±1.33 9 months: 4.35±1.46	Bl: 9.26±1.45 9 months: 5.07±0.86	Bl: 10.07±1.76 9 months: 4.57±1.68
**Defect fill (DF)**			Bl: 5.52±2.97 9 months: 3.47±1.61	Bl: 6.97±3.68 9 months: 5.29±2.63	Bl: 5.93±1.45 9 months: 2.95±1.22	Bl: 6.76±1.56 9 months: 2.90±1.40

## Results

### Forest plots

**Figure 2.**
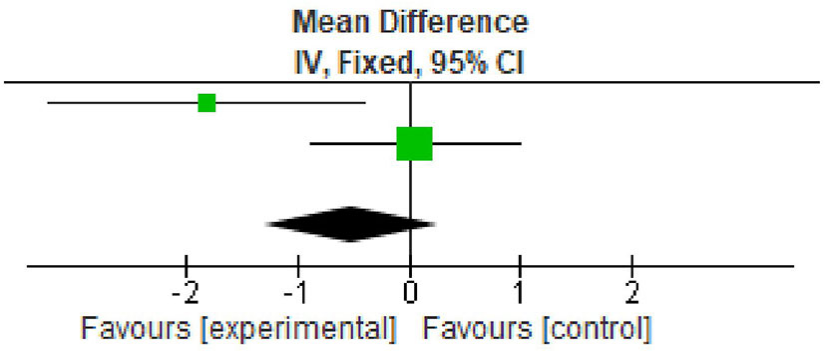
Forest plot of Plaque index.

**Figure 3.**
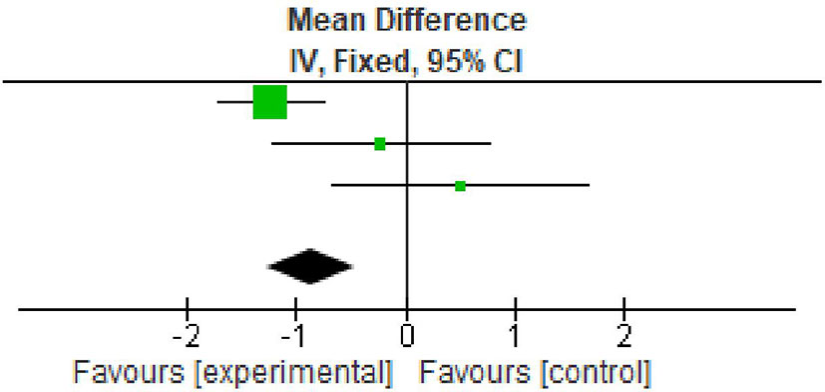
Forest plot of probing pocket depth.

**Figure 4.**
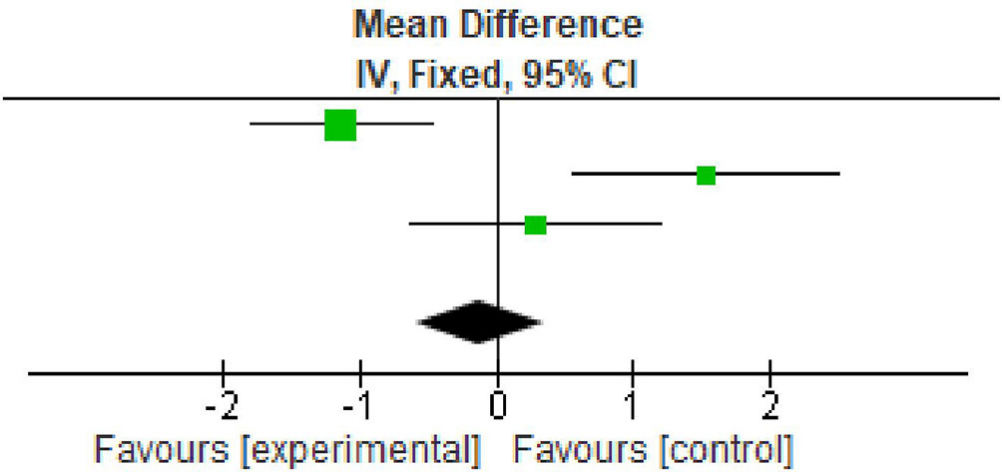
Forest plot of clinical attachment loss.

**Figure 5.**
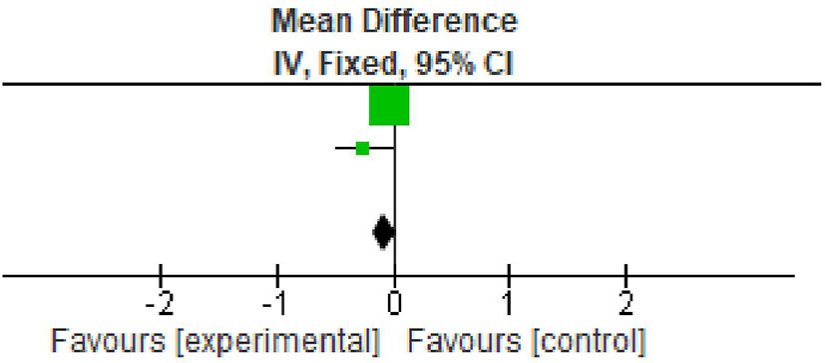
Forest plot of defect fill.

The analysis of the PI, PPD, CAL, DF was done separately for the interventional (OFD+T-PRF) and control groups (OFD alone). The details of the studies included for meta-analysis are given in Figures 2- 5. All the included studies reported the use T-PRF combined with OFD. Three articles reported the use of T-PRF in infrabony defects, ^20^^,^^22^^,^^25^ one study reported use of T-PRF combined with allograft for patients with chronic periodontitis. ^28^ Another study reported its use in sinus lift procedures. ^16^ Two studies reported its use in gingival recession ^21^^,^^29^ and other in healing of extraction socket. ^29^ Based on the overall similarities in participants, outcome and data three studies were found to be eligible for meta-analysis. ^20^^,^^22^^,^^25^ The conclusion regarding the overall effect size estimates were made based on the meta-analysis.

### Plaque index

All the included studies reported PI at baseline and after nine months. ^20^^,^^22^^,^^25^ However, two studies reported data in the form of mean±standard deviation. All the studies showed statistically significant reduction in plaque index at nine months follow up period. The overall meta-analysis showed marginally significant reduction in plaque scores after nine months. (Mean Difference -0.52; 95% CI -1.30 to 0.27; p=0.03; I ^2^ 78%; two studies; 33 participants). However, in view of significant heterogeneity (I ^2^=78%), the results need to be interpreted with caution because of increased heterogeneity as shown in Table 3 and Figure 2.

**Table 3.** Data of plaque index for forest plot.

Study or subgroup	Experimental			Control			Weight	Mean difference
	Mean	SD	Total	Mean	SD	Total		
Gummaluri *et al.* 2020	3.47	1.61	18	5.29	2.63	18	30.3%	-1.82 [-3.24, -0.40]
Ustaoğlu *et al.* 2020	2.95	1.22	15	2.9	1.4	15	69.7%	0.05 [-0.89, 0.99]
Total (95% CI)			33			33	100%	-0.52 [-1.30, 0.27]

Heterogeneity: Chi ^2^=4.61, df=1 (p=0.03); I ^2^=78%.

Test for overall effect: Z=1.29 (p=0.20).

### Probing pocket depth

All the three included studies reported data on PPD at baseline and after nine months. All the studies reported significant reduction in PPD. The overall meta-analysis showed marginally significant reduction in PPD scores after nine months. (MD -0.85; 95% CI -1.26 to -0.45; p=0.01; four studies; 62 participants). However, in view of significant heterogeneity (I ^2^=93%), the results need to be interpreted with caution as shown in Table 4, Figure 3.

**Table 4.** Data of probing pocket depth for forest plot.

Study or subgroup	Experimental			Control			Weight	Mean difference
	Mean	SD	Total	Mean	SD	Total		
Arabaci *et al.* 2018	3.82	0.87	29	5.05	0.99	29	71.2%	-1.23 [-1.71, -0.75]
Gummaluri *et al.* 2020	4.12	1.58	18	4.35	1.46	18	16.6%	-0.23 [-1.22, 0.76]
Ustaoğlu *et al.* 2020	5.07	1.56	15	4.57	1.68	15	12.2%	0.50 [-0.66, 1.66]
Total (95% CI)			62			62	100%	-0.85 [-1.26, -0.45]

Heterogeneity: Chi ^2^=9.11, df=2 (p=0.01); I ^2^=78%.

Test for overall effect: Z=4.13 (p<0.0001).

### Clinical attachment loss

All the included studies reported clinical attachment loss (CAL) gain at baseline and after nine months. All the studies reported significant improvement in CAL gain. The overall meta-analysis showed marginally significant increase in CAL gain after nine months. (MD -0.12; 95% CI - 0.59 to -0.35; p<0.0001; three studies; 62 participants). However the percentage of variation (I ^2^ – 55%) was found to be minimum in all the three included studies, therefore making the interpretation of result favourable as shown in Table 5, Figure 4.

**Table 5.** Data of clinical attachment loss for forest plot.

Study or subgroup	Experimental			Control			Weight	Mean difference
	Mean	SD	Total	Mean	SD	Total		
Arabaci *et al.* 2018	4.01	1.31	29	5.14	1.29	29	49.5%	-1.13 [-1.80, -0.46]
Gummaluri *et al.* 2020	4.82	1.78	18	3.29	1.1	18	23.7%	1.53 [0.56, 2.50]
Ustaoğlu *et al.* 2020	4.46	0.9	15	4.17	1.56	15	26.7%	0.29 [-0.62, 1.20]
Total (95% CI)			62			62	100%	-0.12 [-0.59, 0.35]

Heterogeneity: Chi ^2^=20.72, df=2 (p<0.0001); I ^2^=90%

Test for overall effect: Z=0.50 (p=0.62).

### Defect fill

All the included studies reported defect fill at nine months. However quantitative assessment was only possible for two studies because of difference in reporting of outcomes. ^20^^,^^22^ Significant defect fill was observed in both the studies. The overall meta-analysis showed a marginally significant increase in defect fill after nine months (MD - 0.07; 95% CI -0.17 to -0.03; p=0.09; two studies; 47 participants). Overall defect fill was not statistically significant. However in spite of improvement in defect fill, in view of significant heterogeneity found the results needs to be interpreted with caution as shown in Table 6, Figure 5.

**Table 6.** Data of defect fill for forest plot.

Study or subgroup	Experimental			Control			Weight	Mean difference
	Mean	SD	Total	Mean	SD	Total		
Arabaci *et al.* 2018	0.55	0.22	29	0.58	0.21	29	82.7%	-0.03 [-0.14, 0.08]
Gummaluri *et al.* 2020	0.45	1.36	18	0.71	0.38	18	17.3%	-0.26 [-0.50, -0.02]
Total (95% CI)			47			47	100%	-0.07 [-0.17, 0.03]

Heterogeneity: Chi ^2^=2.87, df=1 (p=0.09); I ^2^=65%.

Test for overall effect: Z=1.36 (p=0.17).

### Risk of bias (ROB) in the studies

**Figure 6.**
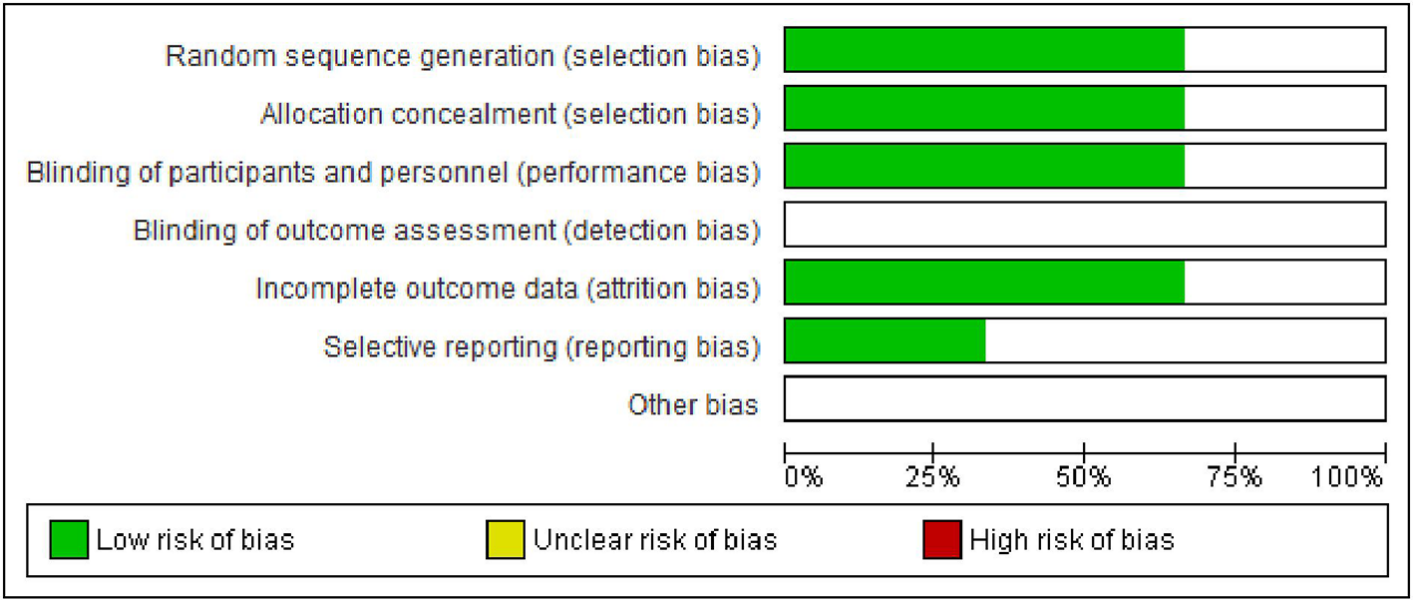
Risk of bias graph.

**Figure 7.**
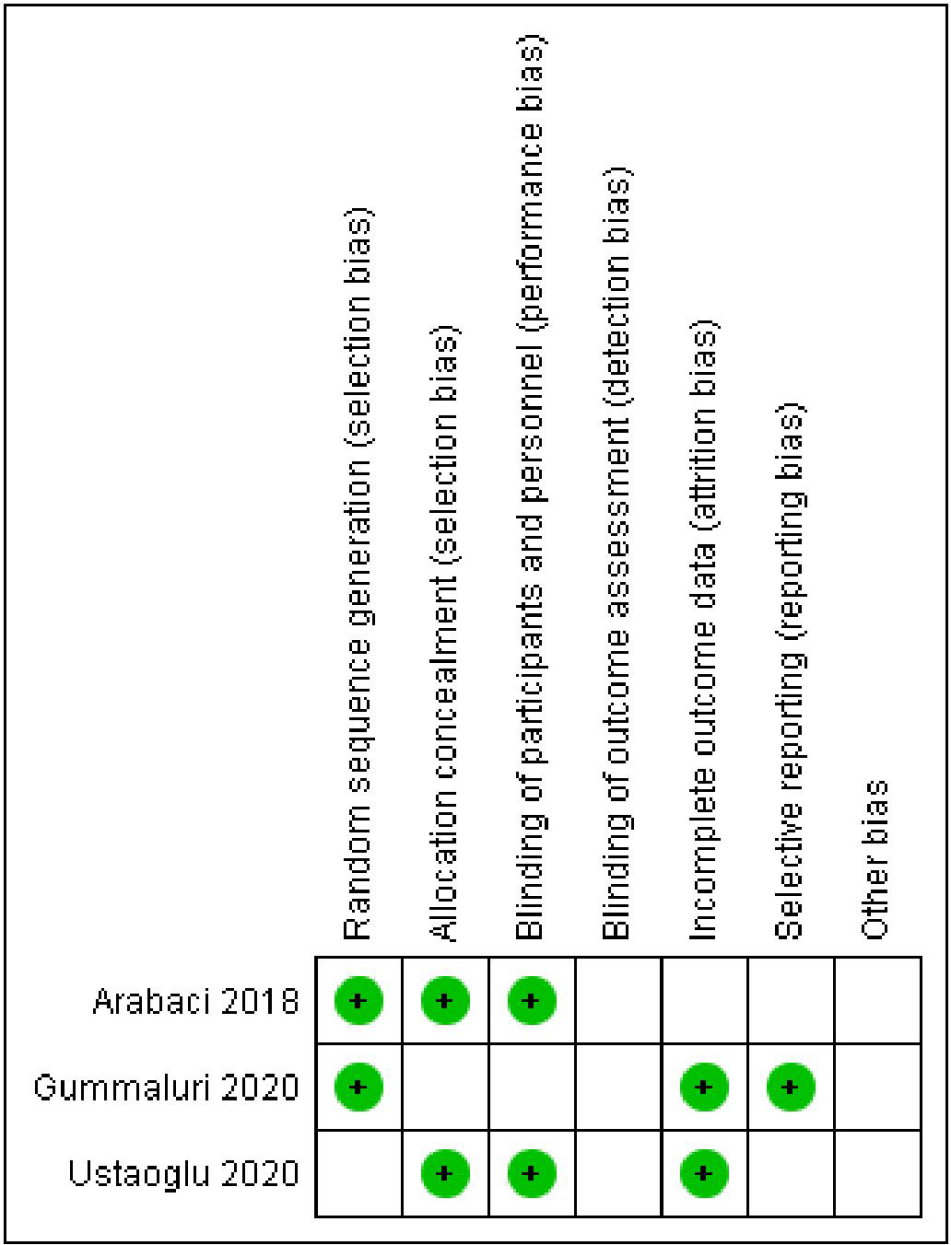
Risk of bias summary.

Allocation: One study reported use of the coin toss method for allocation hence was graded as low risk, ^22^ the other two did not report the method and hence were graded as unclear. ^20^^,^^25^ Blinding: all the studies were double blinded and were kept in low-risk bias. ^20^^,^^22^^,^^25^ Missing result information: discretionary reporting In terms of reporting, we classified all three studies as low risk. The bias in the behaviour or observations: since all procedures and observations were carried out by a single trained professional in each research, we classified the study as low risk in terms of performance or observational bias. Figures 6 and 7 show, respectively, a summary of the risk of bias in the included studies and its graphical depiction.

## Discussion

Autologous platelet concentrates (APCs) are known to improve or accelerate the bone regeneration process. ^20^ Efficacy of plasma fractions with higher platelet concentrations, such as platelet-rich plasma (PRP) and platelet-rich fibrin (PRF) have been successfully linked to connective tissue and bone repair. Evidence from in-vitro studies stated that plasma fractions enhance the quality and quantity of bone defects induced experimentally. ^21^^,^^22^ Since past few years, efforts have been made to enhance the PRFs characteristics and quality. Titanium Platelet Rich Fibrin (T-PRF) is one of them. ^23^ Because silica is no longer contained in the third-generation platelet concentrate, there are no longer any recognised risks associated with using glass vacuum containers to prepare PRF. APCs when used in conjunction with bone substitutes to produce new bone is not backed up by any compelling evidence. However, When T-PRF was used in conjunction with DFDBA during sinus-lifting surgeries, Olgun et al. (2018) observed rapid bone development in 4 months as opposed to 6 months in their clinical, radiological, and histological data. ^24^

There are seventeen pieces of literature published on T-PRF. Two *in-vitro* studies have been performed by Tunali *et al*. ^18^^,^^19^ They discovered a mature fibrin network. In the T-PRF membrane, they discovered islets of bone tissue and newly developing connective tissue. These findings demonstrate that T-PRF could, within 30 days of treatment, cause the development of new bone and connective tissue in a rabbit model of wound healing. They have also described the structural properties of T-PRF, and it was compared to L-PRF. Another *in-vitro* study evaluated the regulation of gingival keratinocyte adherence, dissemination, and cytokine expressions on titanium and PRF surfaces. ^30^ Ustaoğlu *et al.* (2016) ^29^ in their clinical study assessed the T-PRF’s clinical effects on human palatal mucosal wound healing (PMWH), and its impact on “time-dependent variations in palatal soft-tissue thickness (PSTT), a novel idea, was discovered. Uzun *et al.* (2017) ^21^ compared the results of connective tissue graft with autologous T-PRF (CTG). T-PRF (63 teeth) or CTG were utilized to treat 114 Miller Class I/II gingival recessions with abrasion defects utilizing a modified tunnel technique (51 teeth). Olgun *et al.* (2018) ^16^ investigated the differences between using an allograft or totally autologous T-PRF in sinus-lifting surgeries on the clinical, radiological, and histological levels. Arabaci *et al.* (2018) ^20^ T-PRF and open flap debridement (OFD) were examined for their effects on biological markers in gingival crevicular fluid (GCF) and periodontal results. TPRF+OFD or OFD alone were used to treat 29 subjects with chronic periodontitis. Ustaoğlu *et al.* (2019) ^31^ analysis of extraction sockets preserved by L-PRF and T-Fractal PRF’s dimension (FD) and early soft tissue healing characteristics. Ustaoğlu *et al.* ^31^ evaluated the impact of T-PRF and Connective tissue graft (CTG) on peri-implant soft tissue thickness, and crestal bone level. Thirty implants were inserted into 30 patients while the soft tissue was simultaneously augmented with either T-PRF or CTG. Gummaluri *et al.* (2020), ^22^ based on clinical and radiographic criteria, assessed the efficacy of T-PRF and L-PRF in the management of intra-bony defects.

Out of the above-mentioned studies, three studies were included for meta-analysis. ^20^^,^^22^^,^^25^ Results of meta-analysis supported that T-PRF is effective for correction of both hard and soft tissue defects. We included three studies that have reported data from 100 participants, aged 18+ years, that have used T-PRF in infrabony defects treated with open flap debridement. Studies have reported data on periodontal parameters like pocket depth, gingival bleeding, clinical attachment loss. Indices including plaque index and gingival index. Meta analysis was done for PPD, CAL, PI and defect fill parameters. The overall results of meta-analysis suggest that T-PRF is a better alternative to other platelet concentrates for both hard and soft tissue parameters.

## Conclusion

This review focused on answering if T-PRF is a better alternative when compared with other platelet concentrates for periodontal regenerative procedures. The overall qualitative and quantitative analysis suggest that T-PRF have superior structural properties and thicker fibrin network. It provides superior results when used alone or combined with autograft or allograft. However, due to limited number of studies done so far, we recommend that more high quality RCTs to be conducted on this topic.

### Recommendation

This systematic review examines the sufficient clinical and radiographic evidence regarding the regenerative capabilities of T-PRF, either alone or in combination with bone grafts, for both hard and soft tissue procedures. However, there is a need for well-designed and adequately powered randomized controlled trials (RCTs) that demonstrate the clinical and histologic outcomes of T-PRF, in order to fully understand and confirm its potential role in regenerative dentistry.

Future studies should aim to address the current heterogeneity in the literature, particularly in the selection of optimal biological agents that facilitate accelerated bone growth, variations in formulations, investigative methods, and durations of follow-up. Additionally, there should be a focus on emphasizing the application of T-PRF membranes.

From a scientific and clinical perspective, the challenge posed by the barrier concept in guided bone regeneration (GBR) mechanisms is likely to inspire new research inquiries and broaden future clinical opportunities for bone regeneration, particularly in relation to alveolar ridge preservation (ARP).

## Data Availability

Figshare: Prisma Checklist. https://doi.org/10.6084/m9.figshare.22341559.v1. ^32^ Data are available under the terms of the Creative Commons Attribution 4.0 International license (CC-BY 4.0).
