# Supplementary material for: Clinical efficacy of titanium prepared platelet rich fibrin in periodontal regeneration: A systematic review and meta-analysis
Source: F1000Res. 2024 May 24;12:393. Originally published 2023 Apr 13. [Version 2] doi: 10.12688/f1000research.131461.2 (PMC11406120; doi:10.12688/f1000research.131461.2)
Supplement: Peer review history

# Reviewer report: Reviewer response for version 2

**DOI:** 10.5256/f1000research.163418.r320559

**Published:** 2024-09-16

**Authors:** Sarah Al-Rihaymee

**Affiliations:** 1 University of Babylon, Babylon, Iraq

Dear Authors,

 I would like to express my appreciation for your outstanding work on this systematic review and meta-analysis. Your efforts in consolidating the available evidence on the clinical efficacy of titanium-prepared platelet-rich fibrin (T-PRF) in periodontal regeneration are highly commendable.

 Limitation Not Addressed: While the review emphasizes the limited number of included studies, it does not address other critical limitations. For example, challenges related to the preparation and application of PRF, such as its slippery nature, and its short lifespan, with PRF resorbing within approximately two weeks, are not discussed. These factors can substantially influence the clinical efficacy and outcomes of PRF in periodontal regeneration and should be considered in the interpretation of results. Please ensure that the aforementioned comment is reflected in the discussion.

 Your work provides valuable insights into the potential advantages of T-PRF, but acknowledging these additional limitations would enhance the depth and applicability of your findings.

Are the rationale for, and objectives of, the Systematic Review clearly stated?

Yes

Is the statistical analysis and its interpretation appropriate?

Yes

If this is a Living Systematic Review, is the ‘living’ method appropriate and is the search schedule clearly defined and justified? (‘Living Systematic Review’ or a variation of this term should be included in the title.)

Yes

Are sufficient details of the methods and analysis provided to allow replication by others?

Yes

Are the conclusions drawn adequately supported by the results presented in the review?

Yes

Reviewer Expertise:

Immunity, Periodontology, implant, Microbiology

I confirm that I have read this submission and believe that I have an appropriate level of expertise to confirm that it is of an acceptable scientific standard.

## References

[1] : The efficacy of non-surgical platelet-rich fibrin application on clinical periodontal parameters and periostin level in periodontitis: Clinical trial. *J Cell Mol Med* .2023;27(4) : 10.1111/jcmm.17675 529-537 10.1111/jcmm.17675 36691719 PMC9930425

[2] : Platelet-rich fibrin as an adjunct to scaling and root planing in treatment of shallow periodontal pockets: A randomized clinical trial. *J Oral Biosci* .2024;66(3) : 10.1016/j.job.2024.07.002 612-618 10.1016/j.job.2024.07.002 38997108

# Reviewer report: Reviewer response for version 2

**DOI:** 10.5256/f1000research.163418.r282415

**Published:** 2024-09-11

**Authors:** Sara Bernardi

**Affiliations:** 1 Department of Life, Health and Environmental Sciences, University of L'Aquila, L’Aquila, Italy

Manuscript can be now indexed.

Are the rationale for, and objectives of, the Systematic Review clearly stated?

Yes

Is the statistical analysis and its interpretation appropriate?

I cannot comment. A qualified statistician is required.

Are sufficient details of the methods and analysis provided to allow replication by others?

Yes

Are the conclusions drawn adequately supported by the results presented in the review?

Yes

Reviewer Expertise:

Dentistry

I confirm that I have read this submission and believe that I have an appropriate level of expertise to confirm that it is of an acceptable scientific standard.

# Reviewer report: Reviewer response for version 1

**DOI:** 10.5256/f1000research.144303.r219505

**Published:** 2023-11-23

**Authors:** Sara Bernardi, Davide Gerardi

**Affiliations:** 1 Department of Life, Health and Environmental Sciences, University of L'Aquila, L’Aquila, Italy; 2 Universita degli Studi dell'Aquila, L'Aquila, Abruzzo, Italy

Dear authors, congratulations for the study and your contribution to the research. This systematic review and meta-analysis discusses about the efficacy of T-PRF in periodontal regenerative procedures, an interesting topic regarding a specific type of autologous platelet concentrates, thus T-PRF, applied to the widely-studied field of periodontal regeneration. The search strategy is well defined and clear. However, I suggest some revisions in order to improve the quality of the manuscript.

 Introduction: I suggest to deepen the description of APCs, giving a more complete description of the protocol to obtain T-PRF, but also describing the histological characteristics of platelets concentrates, from the cited fibrin network to the other components of PRF.

 Besides, it would be useful to describe the differences between the first and the second generations of autologous platelet concentrates and their application in the different fields of oral surgery and periodontology, focusing on the involvement of T-PRF and periodontal regeneration procedures.

 Discussion: the discussion should me more consistent; the aim of the study is to compare T-PRF to other APCs in periodontal regeneration, so, before comparing the results,  I suggest to add more information, according to the results of the study, about the APCs that in the included studies have been compared to the main subject of discussion, thus the T-PRF.

 Moreover, the different periodontal regenerative techniques compared should be shortly described. These two topics can help to evaluate the advantages and disadvantages of T-PRF, in relation to the other interventions.

 Finally, I suggest to highlight the limits of the study, from which the future perspectives and recommendations, explained in the conclusion, derive.

Are the rationale for, and objectives of, the Systematic Review clearly stated?

Yes

Is the statistical analysis and its interpretation appropriate?

I cannot comment. A qualified statistician is required.

Are sufficient details of the methods and analysis provided to allow replication by others?

Yes

Are the conclusions drawn adequately supported by the results presented in the review?

Yes

Reviewer Expertise:

Dentistry

We confirm that we have read this submission and believe that we have an appropriate level of expertise to confirm that it is of an acceptable scientific standard, however we have significant reservations, as outlined above.

# Response

**Published:** 2024-05-08

**Authors:** Ranu Oza

**Affiliations:** Datta Meghe Institute of Medical Sciences, India

Respected Sir,

 Thank you for you suggestions. 

 Thank you for your insightful feedback on our manuscript. We have incorporated the following revisions based on your suggestions:

1. **Description of APCs and T-PRF Protocol:** We have provided a more comprehensive description of autologous platelet concentrates (APCs) and detailed the protocol for obtaining T-PRF (Titanium-prepared platelet-rich fibrin) in the methods section. This addition aims to enhance clarity and understanding of the experimental procedure.
2. **Discussion on the Use of APCs in Periodontal Regeneration:** In the discussion section, we have elaborated on the aim and practical applications of APCs in periodontal regeneration. By highlighting the role of APCs, we aim to emphasize their significance and potential benefits in clinical settings.
3. **Highlighting Results Related to APCs:** We have specifically emphasized the results related to APCs within the study findings. This ensures that the impact and implications of APCs are prominently discussed and analyzed.
4. **Limitations, Future Perspectives, and Recommendations:** In response to your suggestion, we have highlighted the limitations of our study in the discussion section. These limitations serve as a basis for identifying future perspectives and recommendations, which have been elaborated upon in the conclusion. 

# Reviewer report: Reviewer response for version 1

**DOI:** 10.5256/f1000research.144303.r178213

**Published:** 2023-06-27

**Authors:** Anurag Satpathy

**Affiliations:** 1 Department of Periodontics, Institute of Dental Sciences, Siksha O Anusandhan University, Bhubaneswar, Odisha, India

Authors have carried out SR + MA on" Clinical efficacy of titanium prepared platelet-rich fibrin in periodontal regeneration" 

 There are a few areas which need to be clarified.

**Introduction**: 

- "One of them is titanium....". There seems to be a disconnect between the statement and its previous sentence.
- The objective statement for the SR is not clearly mentioned in the main text. Authors should write the objective statement

**Methods:** 

- Authors should present the Research Question and the PICOT in a clear manner. PROSPERO Number should be mentioned in this section.
- Fig 1: Authors are suggested to use the 2020 PRISMA Checklist and The Flow Diagram

**Results**: 

- Authors should write a short description of the included studies (table 2)
- By Using RevMan, Table 3 and Fig 2, Table 4 and Fig 3, Table 5 and Fig 4, Table 6 and Fig 5 can be combined into one. That is a standard and more appealing way to represent the Forest plots along with associated data. Authors are suggested to change.

**Conclusions:**

- The overall effect for plaque Index, Clinical Attachment Loss was found to be not significant and only Probing Depth was found to be significant, with marginal significance in defect fill with only two studies. Therefore, the conclusion statement should be made with caution.

Are the rationale for, and objectives of, the Systematic Review clearly stated?

Partly

Is the statistical analysis and its interpretation appropriate?

Yes

Are sufficient details of the methods and analysis provided to allow replication by others?

Partly

Are the conclusions drawn adequately supported by the results presented in the review?

Partly

Reviewer Expertise:

Periodontology, Dental  Implantology,  Biofilm Formation, Clinical Dentistry, Periodontal Regeneration, Biomaterials, Quality of Life Research

I confirm that I have read this submission and believe that I have an appropriate level of expertise to confirm that it is of an acceptable scientific standard, however I have significant reservations, as outlined above.

# Response

**Published:** 2024-05-08

**Authors:** Ranu Oza

**Affiliations:** Datta Meghe Institute of Medical Sciences, India

Respected Sir,

 Thank you for your suggestions.

 Thank you for your constructive feedback on our manuscript. We have carefully considered your comments and made the following revisions:

1. **Introduction:** We have thoroughly modified and corrected the introduction to enhance clarity and coherence.
2. **Objective Statement:** The objective statement for the systematic review is now explicitly mentioned in the main text, providing a clear overview of the study's aims.
3. **Research Question and PICOT:** We have rephrased the research question and PICOT (Population, Intervention, Comparison, Outcome, Time) elements in a more concise and understandable manner. Additionally, we have included the PROSPERO registration number in this section for transparency.
4. **PRISMA Checklist and Flow Diagram:** We have updated the PRISMA (Preferred Reporting Items for Systematic Reviews and Meta-Analyses) Checklist to the 2020 version and included the updated Flow Diagram to accurately reflect our review process.
5. **Results Section:** Due to the generation of forest plots and tables using Review Manager, we were unable to combine them into a single figure. However, we have ensured that both formats are presented clearly for better visualization and interpretation.
6. **Description of Included Articles:** A concise description of the included articles has been added to provide readers with a brief overview of each study's characteristics.

We believe that these revisions have strengthened the manuscript and improved its overall quality.
